# Gut Microbiota Mediates the Relationship Between Immune Cells and Familial Hypercholesterolemia: A Mendelian Randomization Study

**DOI:** 10.2174/0118715303441505260410094518

**Published:** 2026-04-30

**Authors:** Shuwei Liang, Zeyu Zhou, Siming Wang, Qian Xue, Aoni Wu, Haopeng Yang, Qiyu Sun

**Affiliations:** 1 Department of Clinical Laboratory, Affiliated Hospital of Chengde Medical College, Chengde, Hebei Province, China;; 2 Hebei Key Laboratory of Panvascular Diseases, Chengde, Hebei Province, China

**Keywords:** Familial hypercholesterolemia, immune cell phenotypes, gut microbiota, mendelian randomization analysis, mediation analysis, low-density lipoprotein

## Abstract

**Introduction:**

Although extensive studies have revealed the critical role of immune cells in the pathogenesis of Familial Hypercholesterolemia (FH), the interplay between immune cells and gut microbiota and their potential mediating effects in developing this disease still lacks systematic investigation. This study aimed to investigate the potential causal effects of immune cells on FH and evaluate the mediating role of gut microbiota in this process.

**Methods:**

This study employed a two-sample Mendelian Randomization (MR) analysis to assess the causal relationships among immune cells, gut microbiota, and FH. Instrumental variables were selected as single-nucleotide polymorphisms significantly associated with exposures from relevant Genome-Wide Association Studies (GWAS). The Inverse-Variance Weighted (IVW) method was primarily used for causal effect estimation, supplemented by MR-Egger and weighted median methods to validate the robustness of the results. The validity of instrumental variables was ensured through heterogeneity tests (Cochran’s Q) and pleiotropy analysis (MR-Egger intercept). Data were sourced from public GWAS databases, ensuring sample independence.

**Results:**

This study identified 34 immune cell traits and 12 gut microbiota taxa significantly associated with FH. Among these, 19 immune cells were inversely associated with FH risk, while 15 showed positive associations; among the gut microbiota, six were inversely associated with FH risk, and the other six were positively associated. Mediation analysis results indicated that the associations between immune cells and FH were partially mediated by five gut microbial taxa (including two phyla, three families, and three genera). Specifically, CD39^+^ activated regulatory CD39^+^ activated Treg %CD4 Treg, CD39 on CD39+ secreting Treg, and CD39 on CD39+ CD4 exerted significant positive mediating effects on FH through *Species Bifidobacterium kashiwanohense*, with mediation proportions reaching 19.9%, 15.7%, and 14.3%, respectively; HLA DR on plasmacytoid DC and HLA DR on CD33- HLA DR^+^ exerted adverse mediating effects on FH through *Family Fibrobacteraceae*, with mediation proportions of -10.2% and -8.95%, respectively.

**Discussion:**

This study provides evidence linking specific immune cell phenotypes and gut microbial taxa to FH, with partial mediation through immune–microbiota interactions. Although FH is primarily a monogenic disorder, these findings suggest that immune and microbial factors may act as modifiers of lipid metabolism and inflammatory burden. The observed mediation effects were modest, indicating complementary rather than dominant roles, and highlight immune–microbial crosstalk as a relevant context for understanding systemic metabolic phenotypes.

**Conclusion:**

This study reveals potential causal links between immune cells and the gut microbiota in FH, providing novel perspectives on the pathogenesis of this disease and potentially offering a theoretical basis for developing targeted therapeutic strategies.

## 1 INTRODUCTION

Familial Hypercholesterolemia (FH) is an autosomal dominant disorder characterized by impaired Low-Density Lipoprotein (LDL) metabolism [1]. This disease primarily stems from pathogenic variants in three key genes: the LDL receptor gene [2], the apolipoprotein B gene [3], and the proprotein convertase subtilisin/kexin type 9 gene [4, 5]. These mutations significantly impair LDL clearance, leading to pathologically elevated LDL cholesterol levels in the circulation [6].

Immune cells are the core components of the immune system, including innate immune cells (*e.g*., macrophages, neutrophils) and adaptive immune cells (*e.g*., T cells, B cells), which maintain organismal homeostasis by recognizing and eliminating pathogens [7]. They function synergistically through cytokine secretion, phagocytosis, and antigen presentation. Research has demonstrated that immune cells play an essential role in the progression of FH. For instance, infiltration of T and B lymphocytes is observed in FH lesions [8]. Th1 cells exacerbate inflammation by secreting pro-inflammatory cytokines like IFN-γ, while Th2 cells, although capable of inhibiting IFN-γ, can upregulate pro-atherogenic factors such as VCAM-1 [9, 10]. Th17 cells promote inflammation *via* IL-17, whereas Treg cells exert protective effects, although oxLDL can impair their function [11, 12].

The gut microbiota is a complex microbial community colonizing the human intestine, primarily composed of bacteria, archaea, fungi, and viruses, involved in host physiological processes such as digestion, immune regulation, and metabolism. Factors including diet, antibiotics, and host genetics influence its composition. Recent advances in metagenomic technologies have significantly propelled gut microbiota research [13-15]. Recent studies suggest that the gut microbiota may affect lipid metabolism in FH patients by modulating bile acid metabolism and cholesterol absorption [16]; for example, specific genera (*e.g*., *Bacteroides*) can promote bile acid transformation, reducing intestinal cholesterol absorption. It has also been found that the gut microbiota of FH patients may exhibit enhanced pro-inflammatory functions, thereby promoting dyslipidemia [17].

However, disentangling causal relationships among immune dysregulation, gut microbiota alterations, and FH remains challenging, as these factors are highly interrelated and subject to confounding and reverse causation. The validity of MR depends on three core assumptions: the genetic instruments must be robustly associated with the exposure of interest, independent of confounders, and affect the outcome exclusively through the exposure. When these assumptions are reasonably satisfied, MR provides a powerful complement to conventional observational analyses. Beyond traditional cardiometabolic traits, MR has been increasingly applied to complex and non-classical exposures, such as behavioral factors. For example, sleep duration has been causally linked to depression risk using MR-based designs, illustrating its applicability to multifactorial phenotypes [18].

In parallel, MR has been successfully incorporated into multi-omics research frameworks to elucidate the mechanistic pathways underlying metabolic disorders, including studies that integrate genetic, microbial, and metabolic data to explore the etiology of hyperuricemia [19]. Such integrative strategies provide a conceptual foundation for investigating immune-microbiota interactions within a causal inference framework.

Building upon this conceptual framework, we first employed a two-sample MR approach to evaluate the causal effects of immune cell phenotypes and gut microbiota on FH. In parallel, a bidirectional MR strategy was implemented to examine potential reverse causal relationships between FH and both immune cell phenotypes and gut microbiota, thereby strengthening inference regarding causal directionality. Bidirectional MR analyses have been widely adopted to clarify directionality in complex disease associations, as illustrated by oncology studies reporting a non-causal relationship between body weight and breast cancer risk [20]. This bidirectional perspective is particularly relevant in the context of FH, where disease status itself may exert downstream effects on immune cell composition and gut microbiota structure. Subsequently, we innovatively constructed a mediation analysis model within the MR framework to determine whether gut microbiota mediates the association between immune cells and FH, thereby integrating immune and microbial components into a unified causal pathway [21].

## 2 MATERIALS AND METHODS

### 2.1 Study Design

This study focused on three core objectives, following a predefined causal sequence from exposures to outcomes and subsequent mediation analysis [22]: (1) To systematically evaluate the forward causal effects of immune cell traits and gut microbial taxa on FH, including the effects of 731 immune cell traits on FH, the effects of 473 gut microbial taxa on FH, and the causal relationships between immune cell traits and gut microbial taxa. (2) To investigate the reverse causal effects of FH on immune cell traits and gut microbial taxa, thereby assessing whether FH status influences immune cell phenotypes and gut microbiota composition. (3) To further elucidate whether gut microbiota acts as a mediator in the causal pathway linking immune cell traits to FH risk.

All analyses were conducted in a stepwise manner consistent with this exposure-outcome-mediation framework. The study strictly adhered to the STROBE-MR guidelines to ensure transparency and reproducibility in study design, data sources, instrumental variable selection, and statistical analyses. For causal inference in the exposure–outcome MR analyses, the Inverse-Variance Weighted (IVW) method served as the primary analytical approach, with immune cell traits or gut microbial taxa specified as exposures and FH as the outcome, and was supplemented by MR-Egger regression, weighted median, weighted mode, and simple mode methods for cross-validation to enhance robustness. To further assess result reliability and consistency, Cochran’s Q test was applied to evaluate heterogeneity, the MR-Egger intercept test to assess directional pleiotropy, the MR-PRESSO method to identify and correct for potential outliers, and leave-one-out analysis to examine the influence of individual Single-Nucleotide Polymorphisms (SNPs) on overall results.

Subsequently, mediation MR analyses were performed by integrating genetically predicted immune cell traits (exposures), gut microbial taxa (mediators), and FH (outcome), thereby extending the primary causal findings into a mechanistic framework.

### 2.2 Mendelian Randomization Assumptions

MR analysis requires fulfillment of three core assumptions: (1) genetic variants serving as instrumental variables must be significantly associated with the exposure; (2) these variants must be independent of all potential confounders; (3) their effect on the outcome must be exclusively mediated through the exposure variable, not *via* alternative pathways (Fig. **1**) [23]. All data were sourced from two authoritative public genomic databases: the FinnGen research project and the OpenGWAS platform. Original data underwent standardized processing and anonymization before upload, with all personally identifiable information removed. Only publicly accessible summary statistics were utilized, which had received ethical approval from relevant institutions and were freely available for research.

### 2.3 Data Sources

#### 2.3.1 FH GWAS Data Source

FH Genome-Wide Association Study (GWAS) data were obtained from the European cohort of the Finnish FinnGen project (Release R12), comprising 6,454 FH cases and 420,066 healthy controls. This dataset was used as the outcome source in all MR analyses. FinnGen is a large population-based biobank study integrating genetic and health registry data from Finnish participants. Project details are accessible *via* the official portal (https://www.finngen.fi/en/access_results).

#### 2.3.2 Immune Cell GWAS Data Source

Summary statistics for immune cell trait GWAS were sourced from the UK Biobank *via* the IEU OpenGWAS platform (https://gwas.mrcieu.ac.uk/). These data include 731 immune cell phenotypes and were used as exposure variables in the primary MR analyses. The dataset was publicly released in 2020 and is available under accession numbers GCST90001391–GCST90002121.

#### 2.3.3 Gut Microbiota GWAS Data Source

Gut microbiota GWAS data were obtained from the European Bioinformatics Institute (EBI) GWAS database (https://www.ebi.ac.uk/gwas/). These data were derived from 5,959 Finnish individuals and were used as exposures in gut microbiota-FH MR analyses and as mediators in subsequent mediation MR analyses. A total of 473 microbial taxa were included, covering multiple taxonomic levels: 10 phyla, 18 orders, 24 classes, 58 families, 143 genera, 213 species-level taxa, and seven unclassified groups. All datasets are registered under accession numbers GCST90032172–GCST90032644 [24].

### 2.4 Instrumental Variable Selection

Given the typically modest association strength of gut microbiota–related SNPs, a relaxed and trait-adaptive selection strategy was adopted. Specifically, to ensure a sufficient number of instrumental variables and avoid unstable causal estimates driven by single-variant instruments, we required at least three independent SNPs per microbial trait and applied a significance threshold *p* < 5 × 10^−6^, depending on the availability of associated variants for each trait. This range reflects a commonly used compromise in microbiome MR studies, where genome-wide significant loci are often sparse, and similar thresholds have been employed in prior literature to enhance instrument representativeness and analytical robustness [25, 26]. For immune cell phenotypes, the conventional threshold of *p* < 5×10^−8^ was used.

SNPs were clumped at r^2^ < 0.001 within a 10,000 kb window to minimize linkage disequilibrium. Instrument strength was evaluated using F-statistics (F > 10) to limit bias from weak instruments (Supplementary Document 1) [27]. To mitigate potential sample overlap, we performed bidirectional MR analyses with separate sets of independent SNPs for the forward (exposure → outcome) and reverse (outcome → exposure) directions, preventing any single SNP from affecting both traits. Using distinct data sources for exposures and outcomes further reduces bias from overlapping samples. Finally, palindromic SNPs were removed after extracting data from IEU OpenGWAS and FinnGen to ensure correct alignment of effect alleles.

In mediation MR analyses, traits showing evidence of reverse causality—where the outcome appeared to causally influence the exposure—were excluded to preserve the assumed causal direction from immune cell traits to gut microbiota and ultimately to FH. This step aligns with the exclusion restriction assumption of MR, which requires that instrumental variables influence the outcome solely through the exposure and not *via* alternative pathways. By removing traits with significant reverse effects, the mediation analysis more accurately reflects the forward causal pathway of interest and minimizes potential bias. A Bonferroni correction was applied to control for multiple testing, with corrected *p* < 0.05 considered statistically significant. Associations that did not reach this threshold but showed *p* < 0.05 without correction were treated as nominal associations, meaning they suggest a potential relationship but require cautious interpretation as they may include false positives due to multiple comparisons.

### 2.5 Statistical Analysis

Analyses were conducted in R (version 4.5.0) using the MRPRESSO (v1.0) and TwoSampleMR (v0.6.8) packages. Five complementary MR methods were integrated to comprehensively assess exposure-outcome causality, with IVW as the core framework. In the primary MR analyses, statistical significance was determined using Bonferroni-corrected thresholds, with corrected *p* < 0.05 considered statistically significant, where the correction factor was based on the number of tested traits at each analytical stage. Associations not surviving this correction were treated as suggestive and were not advanced to downstream analyses. Reliability was ensured through multiple sensitivity analyses: 1) Cochran's Q test identified instrument heterogeneity (*p* < 0.05, indicating significant heterogeneity); 2) MR-Egger intercept and MR-PRESSO global tests detected horizontal pleiotropy (*p* > 0.05, indicating no evidence of directional pleiotropy); 3) Leave-one-out analysis evaluated individual SNP influence. A two-step MR framework was constructed: first, quantifying the total effect of immune cells on FH (βtotal), then estimating path-specific effects for immune cells → gut microbiota (β1) and gut microbiota → FH (β2). Only associations that met the Bonferroni-corrected significance threshold (corrected *p* < 0.05) in the primary MR stage were carried forward to the mediation analyses, thereby ensuring consistency in multiple-testing control across analytical steps. Mediation effects were calculated as the product (β1 × β2), and direct effects as βtotal - (β1 × β2). For mediation MR, statistical significance was defined as corrected *p* < 0.05, ensuring stringent control of false-positive findings (Fig. **2**).

## 3 RESULTS

### 3.1 Causal Effects of Immune Cell Phenotypes on FH

Using the IVW method as the primary analysis, we systematically evaluated causal relationships between 731 immune cell phenotypes and FH. Overall, 34 phenotypes showed significant associations, comprising 15 protective and 19 risk-associated traits, indicating both inhibitory and promoting roles in FH pathogenesis (Fig. **3**).

Protective phenotypes were primarily B cell subsets, such as B cell % CD3− lymphocyte (OR = 0.928; 95%CI:0.888–0.969; *p* < 0.001) and CD24+ CD27+ %B cell (OR = 0.929; 95%CI:0.886–0.974; *p* = 0.0022), while risk phenotypes included CD80 on myeloid DC (OR = 1.062; 95%CI:1.020–1.106; *p* = 0.0035) and HLA DR on CD33-HLA DR+ (OR = 1.056; 95%CI:1.037–1.076; *p* < 0.001).

These associations were consistent across multiple MR methods (weighted median, MR-Egger, simple mode, weighted mode), and quality checks confirmed no significant heterogeneity or horizontal pleiotropy (Supplementary Tables **S1–S2**; Supplementary Figure **1**).

Reverse MR analyses indicated that FH did not significantly affect most of these immune cell phenotypes, with only four subsets showing nominal negative correlations (CD80 on myeloid DC, HLA DR+ NK AC, CD24+ CD27+ %B cell, and Naive CD8br %T cell; *p* < 0.05). This pattern supports the assumed causal direction from immune cells to FH for the majority of traits (Supplementary Table **S3**).

### 3.2 Causal Effects of Gut Microbiota on FH

Two-sample MR analysis identified 12 gut microbial taxa with significant causal associations with FH, including two phyla, two classes, four families, two genera, and two species, showing both protective and risk effects (Fig. **4**). Overall, Family Fibrobacteraceae exhibited a protective effect, while six taxa—including *Class Clostridia*, *Families DTU024 sp002411105* and *CAG-145 sp000435615*, *Genus Faecalicatena sp000364245*, and *Species CAG-269 sp001915995* and *CAG-274 sp000432155*—were associated with increased FH risk.

Representative effect estimates illustrate these patterns: *Family Fibrobacteraceae* (OR = 0.645; 95%CI:0.438–0.950; *p* = 0.0265) and *Class Clostridia* (OR = 1.577; 95%CI:1.202–2.070; *p* = 0.0010). These associations were consistent across supplementary MR methods (weighted median, MR-Egger regression, etc.), confirming the IVW results (Supplementary Figure **2**). Quality assessments further supported robustness: 1) heterogeneity tests were non-significant (Supplementary Table **S4**); 2) no evidence of horizontal pleiotropy was detected (Supplementary Table **S5**).

Bidirectional MR analyses were performed to examine potential reverse causality. Most taxa (9/12) showed no significant effect of FH (all *p*>0.05), while three taxa—*Family CAG-145 sp000435615*, *Phylum Actinobacteriota*, and *Species CAG-269 sp001915995*—demonstrated strong negative causal effects. These findings suggest that FH may selectively influence the colonization of certain commensal microbiota with metabolic regulatory functions, possibly *via* bile acid metabolism or systemic inflammatory changes (Supplementary Table **S6**).

### 3.3 Gut Microbiota Mediates the Effects of Immune Cells on FH

Phenotypes with potential reverse causality—where the outcome might causally influence the exposure—were excluded to preserve the assumed causal direction from immune cell traits to gut microbiota and ultimately to FH, ensuring that the mediation analysis reflects forward causal pathways and avoids bias introduced by reverse effects. Using the selected immune cell phenotypes and gut microbial taxa, a two-step MR approach assessed potential mediation. Specifically, thirty immune cell phenotypes served as exposures and nine gut microbial taxa as outcomes in MR analyses of immune cells → gut microbiota (Fig. **5**). Results revealed significant associations between six immune cell phenotypes and five gut microbial taxa (spanning one phylum, three families, and one genus). For example, CD28 on CD39+ secreting Treg was inversely associated with *Species Bifidobacterium kashiwanohense* (OR = 0.933, 95%CI:0.876–0.993; *p* = 0.0285), while HLA DR on CD33− HLA DR+ was positively associated with *Phylum Actinobacteria* (OR = 1.024, 95%CI:1.007–1.041; *p* = 0.0065), illustrating the directionality of these effects.

Further analysis indicated that multiple immune phenotypes regulated individual microbial taxa. Notably, *Species Bifidobacterium kashiwanohense* was negatively regulated by CD28 on CD39+ secreting Treg (OR = 0.933; 95%CI:0.876–0.993; *p* = 0.0285), CD39+ activated Treg %CD4 Treg (OR = 0.962; 95%CI:0.935–0.990; *p* = 0.0088), CD39 on CD39+ CD4+ (OR = 0.971; 95%CI:0.947–0.996; *p* = 0.0229), and CD39 on CD39+ secreting Treg (OR = 0.972; 95%CI:0.948–0.996; *p* = 0.0224). *Family Fibrobacteraceae* was positively regulated by HLA DR on plasmacytoid DC (OR = 1.009; 95%CI:1.003–1.014; *p* = 0.0022) and HLA DR on CD33-HLA DR+ (OR = 1.011; 95%CI:1.004–1.019; *p* = 0.0033). *Phylum Actinobacteria* was positively regulated by HLA DR on plasmacytoid DC (OR = 1.016; 95%CI:1.003–1.029; *p* = 0.0135) and HLA DR on CD33-HLA DR+ (OR = 1.024; 95%CI:1.007–1.041; *p* = 0.0065). These patterns suggest possible underlying mechanisms whereby immune cells may influence FH risk through modulation of gut microbial communities, potentially affecting cholesterol metabolism, bile acid transformation, and systemic inflammatory responses.

Heterogeneity and pleiotropy analyses confirmed the robustness of these findings, supporting that the observed associations are unlikely to be driven by confounding or invalid instruments (Supplementary Tables **S7–S8**).

### 3.4 Mediation Analysis of Immune Cells, Gut Microbiota, and FH

Mediation analysis was performed after identifying key mediators influencing FH and the effects of immune phenotypes on these mediators. Specifically, we first estimated the impact of immune phenotypes on gut microbiota (β1), then assessed the mediating effect of gut microbiota on FH development (β2) using the same method. Overall, four gut microbial taxa (one phylum, two families, and one genus) were found to significantly mediate the relationship between immune phenotypes and FH (Table **1**).

Positive mediation effects, suggesting that certain immune phenotypes may increase FH risk through specific microbial taxa, were observed for CD39+ activated Treg %CD4 Treg, CD39 on CD39+ secreting Treg, and CD39 on CD39+ CD4+ *via* *Species Bifidobacterium kashiwanohense.* These effects may reflect mechanisms through which immune regulation shapes gut microbial composition, subsequently influencing lipid metabolism and systemic inflammation. The mediation proportions were 19.9% (95%CI: 3.61–36.2%), 15.7% (95%CI: 1.49–30%), and 14.3% (95%CI: 1.29–27.4%), respectively, with β values indicating the magnitude of the mediated effects.

Negative mediation effects, implying that certain immune phenotypes may reduce FH risk through gut microbiota, were observed for CD20 on CD24+ CD27+ *via* *Families DTU024 sp002411105*, and for HLA DR on plasmacytoid DC and HLA DR on CD33− HLA DR+ *via* *Family Fibrobacteraceae* and *Phylum Actinobacteria*. These pathways may reflect mechanisms such as the enhancement of beneficial microbial taxa that improve cholesterol metabolism or reduce systemic inflammation. Effect sizes and mediation proportions were as follows: CD20 on CD24+ CD27+ *via* Families DTU024 sp002411105 (β= -0.00751; 95%CI: -0.0147 to -0.000293; mediation proportion -15.1%), HLA DR on plasmacytoid DC *via* *Family Fibrobacteraceae* (β= -0.00376; 95%CI: -0.00616 to -0.00135; -10.2%) and *via* *Phylum Actinobacteria* (β = -0.00292; 95%CI: -0.00525 to -0.000595; -7.97%), HLA DR on CD33− HLA DR+ *via* *Family Fibrobacteraceae* (β = -0.00488; 95%CI: -0.00814 to -0.00162; -8.95%) and *via* *Phylum Actinobacteria* (β = -0.00436; 95%CI: -0.00753 to -0.0012; -8%).

In summary, these results demonstrate that specific gut microbial taxa mediate both protective and risk-enhancing effects of immune phenotypes on FH, providing mechanistic insights into how immune-microbiota interactions contribute to FH pathogenesis.

## 4 DISCUSSION

This study used multiple MR methods to systematically explore the causal links between immune cell phenotypes, gut microbiota, and FH. The results show that specific immune cell subsets and gut microbiota are key factors in the development of FH, with some providing protective effects and others possibly encouraging disease progression. Mediation analysis uncovered the complexity of the immune-microbial interaction network, confirming that gut microbiota serves as a bridge mediating the influence of immune cells on FH. These findings enhance our understanding of FH pathophysiology and lay a foundation for developing targeted interventions focusing on the immune-microbiota axis.

### 4.1 Immune Contributions to FH Pathogenesis

Our findings provide genetic evidence that immune dysregulation plays a central role in FH pathogenesis. Protective effects were observed for specific B cell subsets, including B cell % CD3− lymphocyte, CD24+ CD27+ %B cell, and CD19 on IgD+ CD24−, suggesting that B cells may help maintain cholesterol homeostasis. Relevant studies indicate that B1 cells produce T cell-independent IgM antibodies with atheroprotective effects, while B2 cells mediate adaptive immunity and differentiate into memory B cells; both subsets can recognize oxLDL, with B1 cells contributing to cardiovascular protection *via* IgM-mediated oxLDL clearance [11].

FH patients also exhibit increased proportions of CD14++CD16+ intermediate monocytes, which show heightened transcription of atherosclerosis-related genes and pro-inflammatory cytokines under oxLDL or inflammatory stimulation [28]. This underscores how immune cell activation and migration, triggered by inflammatory pathways, may intersect with lipid metabolism and exacerbate FH-associated vascular pathology. T cell subsets further illustrate this relationship. Existing research has shown that Th1 and Th17 cells promote vascular inflammation through IFN-γ and IL-17, respectively [29], whereas Tregs generally suppress immune overactivation, although their function may be impaired by oxLDL. Th2 cells, while often anti-inflammatory, can, under certain conditions, upregulate pro-atherogenic factors such as VCAM-1, linking immune signaling to endothelial dysfunction and lipid accumulation [30].

In summary, these results highlight a mechanistic framework in which immune dysregulation contributes to FH by modulating vascular inflammation and lipid handling, identifying potential targets for future immunomodulatory interventions.

### 4.2 Role of Gut Microbiota in FH

Building on the observed effects of immune cell dysregulation on FH, we next examined the potential role of gut microbiota as a complementary layer influencing lipid metabolism and disease risk, highlighting how host immunity and microbial composition may jointly contribute to FH pathogenesis.

This study provides an in-depth investigation into the potential pathogenesis of FH, with particular focus on the association between gut microbiota and disease. Through causal analyses, we identified twelve microbial taxa significantly associated with FH, which can be broadly categorized into two groups: six taxa that potentially increase FH risk and six taxa that demonstrate protective effects. Notably, *Family Fibrobacteraceae* exhibited the most pronounced protective effect (OR = 0.645).

Existing evidence indicates that gut microbiota dysbiosis is a critical contributing factor in hyperlipidemia, largely mediated by microbial fermentation of dietary fiber into Short-Chain Fatty Acids (SCFAs), which participate in metabolic regulatory networks [31]. From a translational perspective, propionate intervention has shown clinically meaningful lipid-lowering effects. Randomized controlled trials demonstrate that exogenous propionate supplementation significantly reduces LDL-C, TC, and non-HDL-C levels, providing direct clinical evidence for microbial metabolites in cholesterol regulation [32].

Mechanistically, recent studies reveal that an 8-week propionate intervention significantly alters bile acid metabolism, increasing serum levels of secondary bile acids such as Deoxycholic Acid (DCA), which negatively correlate with cholesterol levels [33]. Moreover, animal studies indicate that combined administration of propionate with other SCFAs (*e.g*., acetate or butyrate) is more effective than single interventions in activating hepatic LXR and PPARα signaling pathways and promoting the proliferation of beneficial bacterial genera such as Lactobacillus [34].

Overall, these findings suggest that gut microbiota, by modulating short-chain fatty acids and related bile acid metabolism, may influence lipid homeostasis and offer a potential avenue for therapeutic strategies in metabolic disorders such as FH.

### 4.3 Gut Microbiota Mediates the Effects of Immune Cells on FH

To explore mechanisms of immune-microbial interactions, a two-step MR approach identified six immune phenotypes that significantly influence five FH-associated gut microbial taxa. For instance, CD28 on CD39+ secreting Treg showed a significant negative correlation with *Species Bifidobacterium kashiwanohense* (OR = 0.933), indicating a protective effect along this immune–microbial axis. Conversely, HLA DR on CD33− HLA DR+ correlated positively with *Phylum Actinobacteria* (OR = 1.024), suggesting a potential contribution to FH progression through promotion of this phylum. Plasmacytoid Dendritic Cells (pDCs), which bridge innate and adaptive immunity, express HLA DR and play a key role in host-microbe interactions. Beyond antigen presentation alone, HLA DR-dependent activation of pDCs has been shown to influence the intestinal immune environment through regulated secretion of cytokines such as type I interferons, IL-6, and TNF-α, which can alter epithelial barrier function, antimicrobial peptide expression, and nutrient availability, thereby indirectly shaping microbial colonization patterns [35-37]. Such cytokine-mediated modulation provides a more concrete biological basis for how HLA DR+ pDCs may influence taxa, including *Phylum Actinobacteria* and *Family Fibrobacteraceae*, rather than implying direct cell-microbe interactions.

These immune-driven microbial shifts are particularly relevant in the context of mediation analysis, as they provide a biologically plausible link between upstream immune variation and downstream metabolic outcomes. Rather than exerting direct effects on FH, certain immune phenotypes may partially influence FH risk by altering microbial taxa that are themselves causally associated with lipid metabolism, consistent with a mediated causal pathway.

At the same time, his immune-microbial regulation is conceptually aligned with infection-induced immune perturbations observed in *Helicobacter pylori* infection, where epithelial damage and localized immune cell infiltration trigger sustained inflammatory signaling with systemic metabolic consequences [38]. Importantly, these infection models illustrate how immune-driven changes in cytokine profiles and microbial-derived metabolites can propagate beyond local tissues to affect systemic lipid metabolism, supporting the notion that immune activation can indirectly affect metabolic phenotypes through microbe-associated inflammatory pathways.

Finally, we conducted a mediation analysis, which identified four gut microbial taxa that significantly mediated the effects of immune phenotypes on FH. Notably, CD39+ activated Treg %CD4 Treg mediated 19.9% of the total effect on FH *via* *Species Bifidobacterium kashiwanohense*, suggesting that Treg-associated immune regulation may partially translate into metabolic effects through modulation of specific commensal taxa. This finding is consistent with prior evidence that Tregs contribute to maintaining epithelial integrity and anti-inflammatory tone, which can support colonization of beneficial microbes and indirectly influence lipid metabolism [39]. Although the mediation proportion is modest, this is expected in FH, which is mainly caused by monogenic LDLR-pathway defects; these effects are most likely modifier pathways that partially influence metabolic or inflammatory outcomes, rather than primary drivers of the disease [40].

Similarly, HLA-DR+ pDCs mediated −7.97% and −10.2% of FH variation through *Phylum Actinobacteria* and *Family Fibrobacteraceae*, respectively. This mediation is likely to reflect pDCs’ immunomodulatory functions rather than a direct effect on microbial growth. Specifically, HLA-DR-dependent antigen presentation by pDCs can shape downstream T-cell responses, such as the Th17/Treg balance, which in turn influence the local intestinal immune environment and microbial colonization [41, 42]. Additionally, pDCs secrete cytokines including type I interferons (IFN-α/β) and IL-6, which may modulate epithelial barrier integrity, mucus secretion, and local metabolite availability, thereby indirectly affecting microbial abundance. Changes in *Fibrobacteraceae*, a fiber-degrading group, could further influence short-chain fatty acid production, providing a mechanistic link between immune activity, microbial metabolism, and host lipid regulation [43]. While these pathways are supported by experimental and translational evidence, the precise molecular and cellular interactions remain to be empirically validated in the context of FH [44].

In contrast, CD20+ CD24+ CD27+ B cells mediated −15.1% of FH variation *via* *Families DTU024 sp002411105*, suggesting that B-cell–associated immune functions, potentially including mucosal antibody responses or local immune modulation, including IgA-mediated microbial selection and modulation of mucosal metabolic niches, may influence specific microbial taxa that contribute to lipid metabolic balance [45].

Overall, the mediation analysis supports a structured causal framework in which immune phenotypes act upstream, gut microbial taxa function as intermediate mediators, and FH represents the downstream metabolic outcome. Mechanistic interpretation is grounded in established cytokine- and metabolite-mediated pathways rather than speculative direct effects, consistent with the causal resolution afforded by MR.

### 4.4 Limitations and Implications

Although MR offers the advantage of reducing confounding and reverse causation compared with observational studies, several limitations of our study should be considered. First, population stratification may affect the generalizability of our findings. Our analyses are mainly based on European-ancestry cohorts, including FinnGen and the IEU OpenGWAS database. Differences in genetic background, environmental exposures, and lifestyle factors may limit the applicability of these results to non-European or multi-ethnic populations. Second, residual confounding cannot be fully excluded. Although known confounders were controlled, unmeasured factors such as medications or antibiotic history could influence gut microbiota composition and metabolite profiles, potentially affecting microbiota–FH associations. Third, limitations inherent to MR should be noted. While MR offers genetically supported causal inference, effects estimated from germline variants may be attenuated over time due to biological compensation and should not be interpreted as direct estimates of clinical effect sizes.

In summary, these limitations highlight the need for future studies that incorporate multi-ethnic cohorts, longitudinal designs, and multi-omics datasets (including metagenomics, metabolomics, and immune profiling) to further validate our findings and improve their translational relevance in the context of FH.

## CONCLUSION

This study is the first to systematically identify immune cell phenotypes and gut microbial features associated with FH using MR analysis. Notably, specific gut microbes exerted significant mediating effects between immune phenotypes and FH, providing novel insights into FH pathogenesis and a theoretical basis for targeting the immune-microbiota axis in therapy.

## AUTHORS’ CONTRIBUTIONS

The authors confirm their contributions to the paper as follows: S.L.: Contributed to the study conception and design, developed the methodology, validated results, drafted the original manuscript, and was responsible for critical review and editing of the text. Z.Z.: Participated in study design and methodology, contributed to data curation and resource management, performed validation, and was involved in both drafting and revising the manuscript. S.W.: Contributed to the original draft preparation, assisted with data investigation, and supported resource collection. Q.X.: Participated in the original draft writing, contributed to data investigation, and was responsible for data curation. A.W.: Assisted in resource acquisition, data validation, and performed parts of the investigation. H.Y.: Conducted formal statistical analysis and was involved in the validation of results. Q.S.: Contributed to study conceptualization and methodology development, performed data validation and formal analysis, managed resources, drafted and revised the manuscript, and provided overall supervision of the project.

## Figures and Tables

**Fig. (1):**
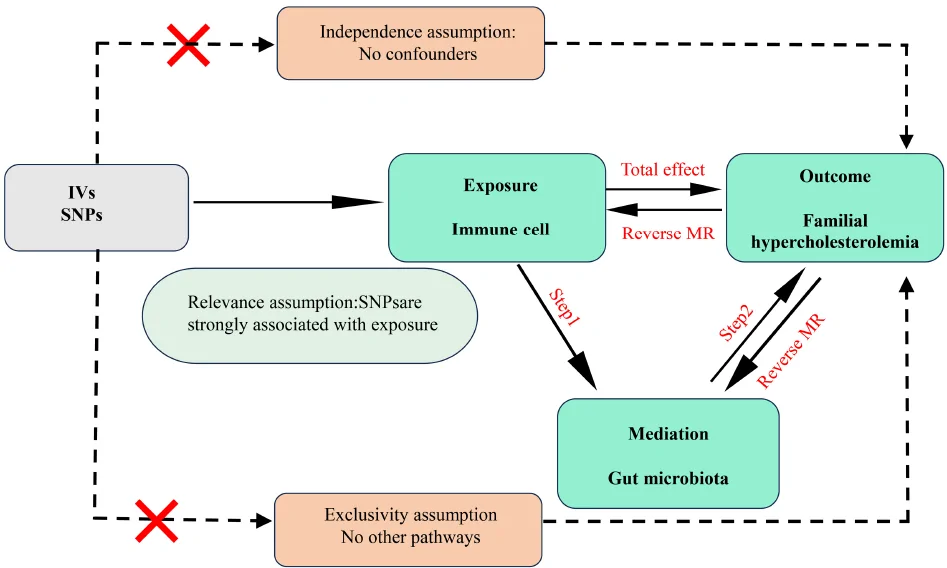
Flow chart of the MR study.

**Fig. (2):**
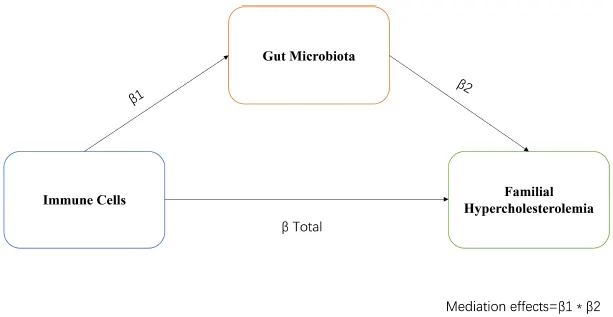
Flow chart of mediation analysis.

**Fig. (3):**
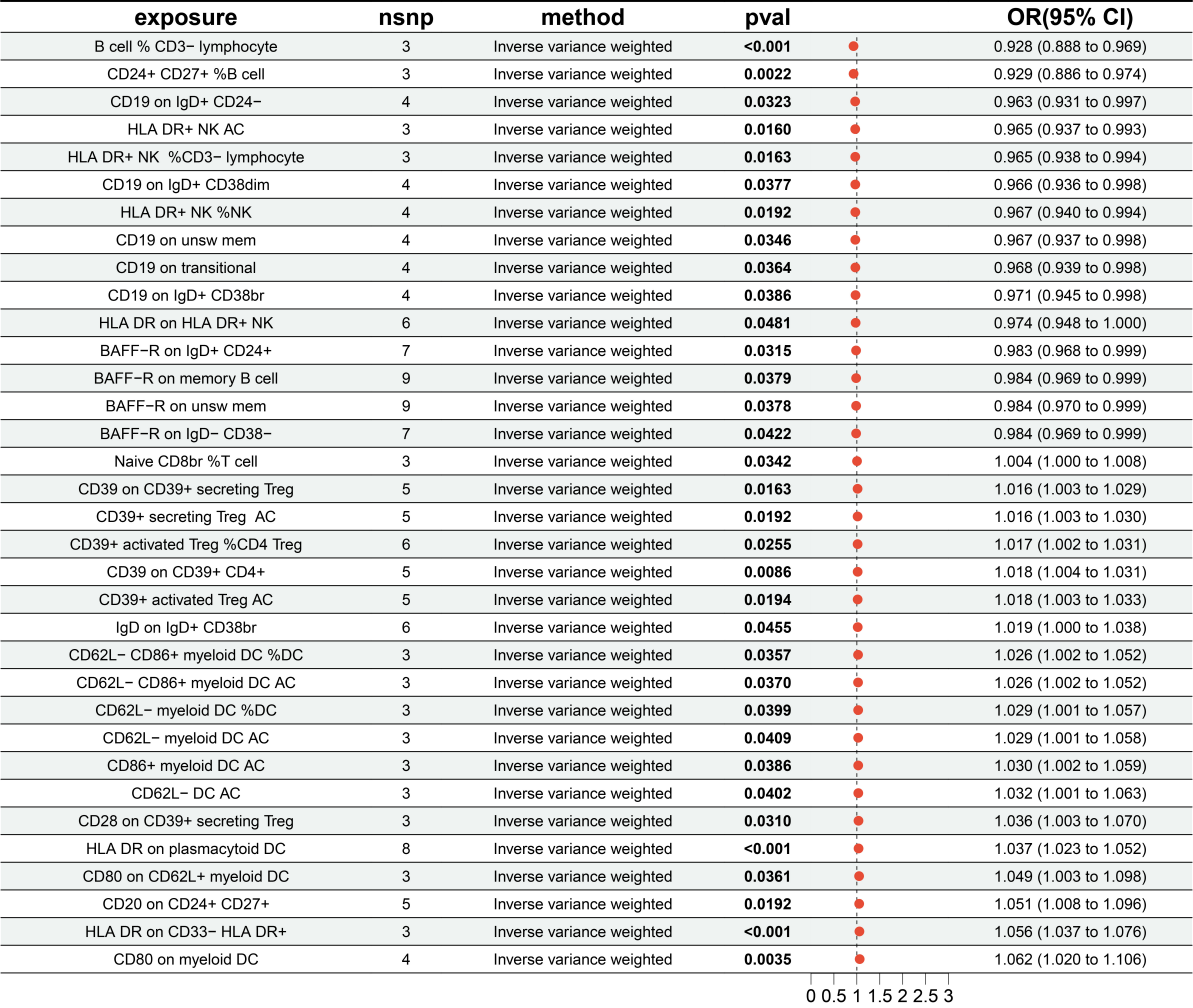
MR forest plot of immune cell phenotypes and FH.

**Fig. (4):**
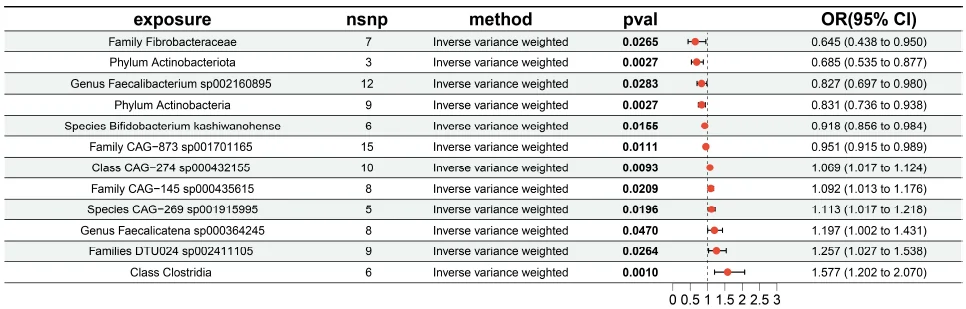
MR forest plot of gut microbiota and FH.

**Fig. (5):**
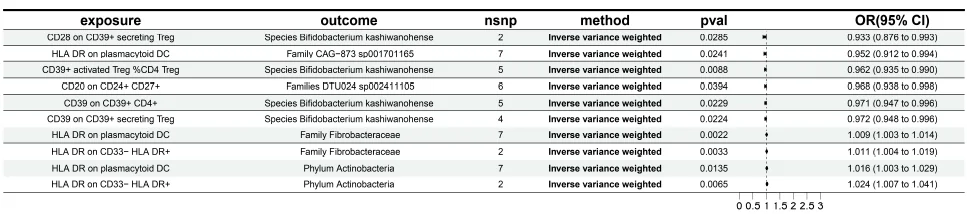
Mediation MR analysis results of gut microbiota.

**Table 1:** Mediation effects and proportions of each gut microbiota.

**Immune Cell**	**Gut Microbiota**	**Outcome**	**Mediated Effect**	**Mediated Proportion**	**Direct Effect**	***p*-value**	
HLA DR on plasmacytoid DC	*Family Fibrobacteraceae*	FH	-0.00376 (-0.00616, -0.00135)	-10.2% (-16.8%, -3.68%)	0.040	0.002
HLA DR on CD33- HLA DR+	*Family Fibrobacteraceae*	FH	-0.00488 (-0.00814, -0.00162)	-8.95% (-14.9%, -2.98%)	0.059	0.003
HLA DR on CD33- HLA DR+	*Phylum Actinobacteria*	FH	-0.00436 (-0.00753, -0.0012)	-8% (-13.8%, -2.19%)	0.059	0.007
CD39+ activated Treg % CD4 Treg	*Species Bifidobacterium kashiwanohense*	FH	0.00328 (0.000594, 0.00597)	19.9% (3.61%, 36.2%)	0.013	0.017
HLA DR on plasmacytoid DC	*Phylum Actinobacteria*	FH	-0.00292 (-0.00525, -0.000595)	-7.97% (-14.3%, -1.62%)	0.040	0.014
CD20 on CD24+ CD27+	*Families DTU024 sp002411105*	FH	-0.00751 (-0.0147, -0.000293)	-15.1% (-29.6%, -0.589%)	0.057	0.041
CD39 on CD39+ secreting Treg	*Species Bifidobacterium kashiwanohense*	FH	0.00248 (0.000234, 0.00472)	15.7% (1.49%, 30%)	0.013	0.030
CD39 on CD39+ CD4+	*Species Bifidobacterium kashiwanohense*	FH	0.0025 (0.000225, 0.00477)	14.3% (1.29%, 27.4%)	0.015	0.031

## Data Availability

The data sets used and/or analysed during this study are available from the corresponding author upon request.

## LIST OF ABBREVIATIONS

FH: Familial Hypercholesterolemia
MR: Mendelian Randomization
IVW: Inverse-Variance Weighted
LDL: Low-Density Lipoprotein
IVs: Instrumental Variables
RCTs: Randomized Controlled Trials
GWAS: Genome-Wide Association Studies
OR: Odds Ratio
β: Effect Sizes
CI: Confidence Interval
SNP: Single Nucleotide Polymorphisms
pDCs: Plasmacytoid Dendritic Cells
DCA: Deoxycholic Acid
SCFAs: Short-Chain Fatty Acids

## STANDARDS OF REPORTING

STROBE-MR guidelines and methodology were followed.
