# Supplementary material for: Gut Microbiota Mediates the Relationship Between Immune Cells and Familial Hypercholesterolemia: A Mendelian Randomization Study
Source: Endocr Metab Immune Disord Drug Targets. 2026 Apr 30;26:E18715303441505. doi: 10.2174/0118715303441505260410094518 (PMC13673436; doi:10.2174/0118715303441505260410094518)
Supplement: Supplementary file 1 [file EMIDDT-26-E18715303441505_SD1.pdf]

Supplementary Material

Gut Microbiota Mediates the Relationship Between Immune Cells and Familial Hypercholesterolemia: A Mendelian Randomization Study

Shuwei Liang[1,2], Zeyu Zhou[1,2], Siming Wang[1,2], Qian Xue[1,2], Aoni Wu[1,2], Haopeng Yang[1,2] and Qiyu Sun[1,2,*]

[1]Department of Clinical Laboratory, Affiliated Hospital of Chengde Medical College, Chengde, Hebei Province, China; [2]Hebei Key Laboratory of Panvascular Diseases, Chengde, Hebei Province, China

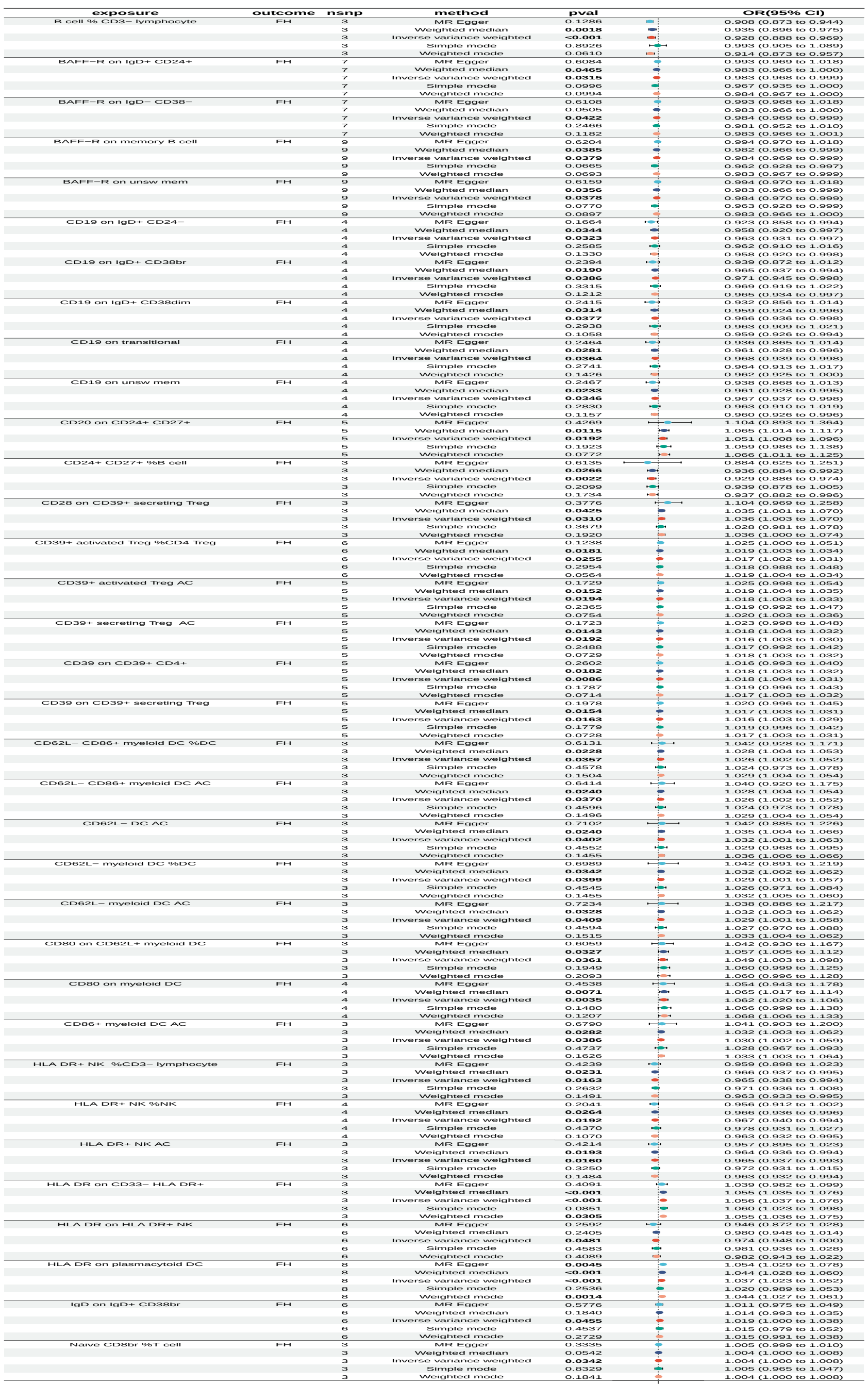

Fig. (S1).

exposure	outcome	nsnp	method	pval	OR(95% CI)
Class CAG−274 sp000432155	FH	10	MR Egger	0.4824	1.069 (0.895 to 1.277)
		10	Weighted median	0.0646	1.063 (0.996 to 1.135)
		10	Inverse variance weighted	**0.0093**	1.069 (1.017 to 1.124)
		10	Simple mode	0.3043	1.062 (0.953 to 1.185)
		10	Weighted mode	0.3103	1.058 (0.955 to 1.172)
Class Clostridia	FH	6	MR Egger	0.5662	1.208 (0.668 to 2.185)
		6	Weighted median	**0.0082**	1.603 (1.130 to 2.273)
		6	Inverse variance weighted	**0.0010**	1.577 (1.202 to 2.070)
		6	Simple mode	0.1022	1.741 (1.011 to 3.000)
		6	Weighted mode	0.0831	1.821 (1.057 to 3.138)
Families DTU024 sp002411105	FH	9	MR Egger	0.2055	1.620 (0.823 to 3.190)
		9	Weighted median	0.2710	1.148 (0.898 to 1.469)
		9	Inverse variance weighted	**0.0264**	1.257 (1.027 to 1.538)
		9	Simple mode	0.6457	1.112 (0.719 to 1.721)
		9	Weighted mode	0.7428	1.078 (0.698 to 1.668)
Family CAG−145 sp000435615	FH	8	MR Egger	0.1587	1.171 (0.966 to 1.421)
		8	Weighted median	0.0635	1.094 (0.995 to 1.204)
		8	Inverse variance weighted	**0.0209**	1.092 (1.013 to 1.176)
		8	Simple mode	0.3483	1.084 (0.926 to 1.268)
		8	Weighted mode	0.2867	1.086 (0.944 to 1.250)
Family CAG−873 sp001701165	FH	15	MR Egger	0.6546	0.980 (0.900 to 1.068)
		15	Weighted median	0.2562	0.972 (0.925 to 1.021)
		15	Inverse variance weighted	**0.0111**	0.951 (0.915 to 0.989)
		15	Simple mode	0.8060	0.988 (0.902 to 1.083)
		15	Weighted mode	0.8157	0.989 (0.902 to 1.084)
Family Fibrobacteraceae	FH	7	MR Egger	0.3784	0.588 (0.200 to 1.726)
		7	Weighted median	0.1381	0.678 (0.405 to 1.133)
		7	Inverse variance weighted	**0.0265**	0.645 (0.438 to 0.950)
		7	Simple mode	0.3200	0.644 (0.291 to 1.427)
		7	Weighted mode	0.3529	0.637 (0.264 to 1.533)
Genus Faecalibacterium sp002160895	FH	12	MR Egger	0.8551	0.959 (0.618 to 1.488)
		12	Weighted median	0.1844	0.856 (0.680 to 1.077)
		12	Inverse variance weighted	**0.0283**	0.827 (0.697 to 0.980)
		12	Simple mode	0.5543	0.886 (0.601 to 1.306)
		12	Weighted mode	0.4893	0.876 (0.609 to 1.259)
Genus Faecalicatena sp000364245	FH	8	MR Egger	0.5092	1.463 (0.506 to 4.229)
		8	Weighted median	0.2034	1.162 (0.922 to 1.463)
		8	Inverse variance weighted	**0.0470**	1.197 (1.002 to 1.431)
		8	Simple mode	0.5160	1.146 (0.776 to 1.693)
		8	Weighted mode	0.5570	1.120 (0.781 to 1.606)
Phylum Actinobacteria	FH	9	MR Egger	0.3681	0.862 (0.638 to 1.166)
		9	Weighted median	**0.0024**	0.781 (0.666 to 0.916)
		9	Inverse variance weighted	**0.0027**	0.831 (0.736 to 0.938)
		9	Simple mode	0.5660	0.909 (0.664 to 1.244)
		9	Weighted mode	**0.0247**	0.779 (0.653 to 0.930)
Phylum Actinobacteriota	FH	3	MR Egger	0.4070	0.417 (0.117 to 1.491)
		3	Weighted median	**0.0083**	0.687 (0.520 to 0.908)
		3	Inverse variance weighted	**0.0027**	0.685 (0.535 to 0.877)
		3	Simple mode	0.4304	0.811 (0.533 to 1.233)
		3	Weighted mode	0.0963	0.637 (0.473 to 0.856)
Species Bifidobacterium kashiwanohense	FH	6	MR Egger	0.7884	0.964 (0.749 to 1.240)
		6	Weighted median	0.1061	0.926 (0.844 to 1.016)
		6	Inverse variance weighted	**0.0155**	0.918 (0.856 to 0.984)
		6	Simple mode	0.5728	0.952 (0.810 to 1.118)
		6	Weighted mode	0.0534	0.843 (0.738 to 0.963)
Species CAG−269 sp001915995	FH	5	MR Egger	0.2334	1.166 (0.953 to 1.426)
		5	Weighted median	0.2129	1.083 (0.955 to 1.227)
		5	Inverse variance weighted	**0.0196**	1.113 (1.017 to 1.218)
		5	Simple mode	0.6928	1.038 (0.875 to 1.231)
		5	Weighted mode	0.7415	1.034 (0.859 to 1.245)

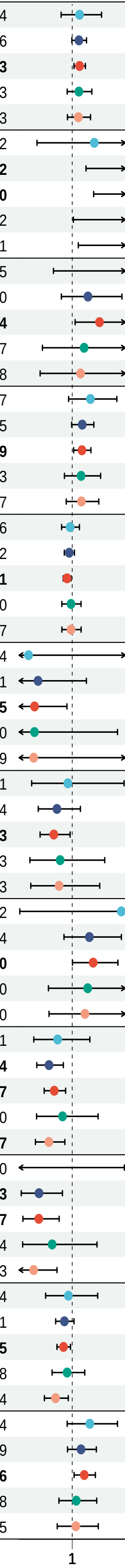

Fig. (S2).
